# Crystal and EM Structures of Human Phosphoribosyl Pyrophosphate Synthase I (PRS1) Provide Novel Insights into the Disease-Associated Mutations

**DOI:** 10.1371/journal.pone.0120304

**Published:** 2015-03-17

**Authors:** Peng Chen, Zheng Liu, Xuejuan Wang, Junhui Peng, Qianqian Sun, Jianzhong Li, Mingxing Wang, Liwen Niu, Zhiyong Zhang, Gang Cai, Maikun Teng, Xu Li

**Affiliations:** 1 Hefei National Laboratory for Physical Sciences at Microscale and School of Life Sciences, University of Science and Technology of China, Hefei, Anhui 230026, People’s Republic of China; 2 Key Laboratory of Structural Biology, Chinese Academy of Sciences, Hefei, Anhui 230026, People’s Republic of China; 3 Department of Otolaryngology Head and Neck Surgery, Fuzhou general hospital of Nanjing Command, PLA, Fuzhou 350025, China; University of Washington, UNITED STATES

## Abstract

Human PRS1, which is indispensable for the biosynthesis of nucleotides, deoxynucleotides and their derivatives, is associated directly with multiple human diseases because of single base mutation. However, a molecular understanding of the effect of these mutations is hampered by the lack of understanding of its catalytic mechanism. Here, we reconstruct the 3D EM structure of the PRS1 *apo* state. Together with the native stain EM structures of AMPNPP, AMPNPP and R5P, ADP and the *apo* states with distinct conformations, we suggest the hexamer is the enzymatically active form. Based on crystal structures, sequence analysis, mutagenesis, enzyme kinetics assays, and MD simulations, we reveal the conserved substrates binding motifs and make further analysis of all pathogenic mutants.

## Introduction

Nucleotides and deoxynucleotides are required for the synthesis of RNA, DNA, and for cofactors such as NADP^+^, FAD^+^, coenzyme A and ATP. The inhibition of the pathways for the biosynthesis of nucleotides blocks transcription and consequently prevents the proliferation of cells. Nucleotides can be synthesized *de novo* or through salvage pathways that use 5-phosphoribosyl 1-pyrophosphate (PRPP) as both a common metabolic intermediate and a regulator [1]. In bacteria and lower eukaryotes, PRPP also serves as a precursor for histidine and tryptophan biosynthesis [2].

PRPP is formed from ATP and D-ribose-5-phosphate (R5P) by the phosphoribosyl pyrophosphate synthetases (PRSs). These enzymes are divided into 3 classes according to their different cofactors dependence. The human PRS family, which has 3 isoforms (hPRS1, hPRS2 and hPRS3), belongs to the class I PRSs that requires divalent metal ions such as Mg^2+^ as the activator, inorganic phosphate ion for activity, and ATP or dATP as the diphosphoryl donor. In addition, they can also be allosterically inhibited by ADP or other nucleotides at the same time [3]. Human PRS isoforms share quite high sequence identity with one another (95.0% between hPRS1 and hPRS2; 94.3% between hPRS1 and hPRS3; and 91.2% between hPRS2 and hPRS3). The hPRS1 and hPRS2 genes are located on the X chromosome and are expressed in a wide range of tissues, but hPRS3 is located on an autosome and is expressed specifically in the testes [4].

Due to the important role of PRPP in the nucleotide metabolic process, the stability of PRPP content is essential for cell physiology and is predominantly controlled by hPRS1, the ubiquitously expressed human PRPP synthetase [5]. Mutations in hPRS1 affect vital cell functions and are related to many diseases and disorders. 7 different single base missense mutations cause hPRS1 superactivity and result in hyperuricemia and gout [6]. Another 8 different single base missense mutations cause insufficient hPRS1 activity and lead to CMTX5 syndrome [7], ARTS syndrome [8] and DFN2 syndrome [9]. 1 novel missense mutation, resulting in the mixed symptoms mentioned above, was reported in 2011 [10]. In addition, 2 missense mutations were reported to cause breast cancer and 1 missense mutation caused colorectal cancers in a study published in 2006 without detailed PRS1 activity or syndromes descriptions [11–14].

The enzymatic activity of hPRS1 is regulated by cofactors, metabolites, and interacting proteins. Inorganic phosphate ions regulate hPRS1 activity via allosteric competition with ADP, which is the most powerful small molecular inhibitor of hPRS1. Inorganic phosphate also promotes the accessibility of the active site by stabilizing a flexible loop involved in ATP binding [4]. Bivalent metal cations, most potently Mg^2+^, are indispensable to forming the Mg-ATP complex that is the actual substrate of the enzyme [15]. In addition, the activity of hPRS1 is regulated by interacting with the 39 kDa and 41 kDa phosphoribosyl pyrophosphate synthetase-associated proteins (PAP39 and PAP41), the two of which are reported to be negative regulators [16]^,^[17].

The crystal structures of PRS and its homolog from *Bacillus subtilis* [18] and other prokaryote have provided the interaction information between PRS and different ligands. The research on hPRS1[4] also revealed a novel allosteric site. However, the underlying molecular understanding of those mutations that cause human diseases are still unclear. Here, using a hybrid structural approach involving sequence conservation analysis, structure comparison and enzymology, we defined the key motifs such as the β2–3 ATP binding motif, the β9–10 R5P binding motif, and the flexible loop for ATP cut. Our results not only confirmed the previous results but also shed additional light on the possible pathogenic mechanism of all 16 mutants.

## Experimental Procedures

### Gene cloning, protein expression and purification

The human PRS1 gene was amplified from the human cDNA library, and the primers used for cloning were synthesized by Invitrogen (Shanghai, China). PRS1 gene was cloned into the NdeI and XhoI sites of a pET22b(+) vector (Novagen). Then, the plasmid was transformed into *Escherichia coli* BL21(DE3) (Merck) for protein expression. Cells were harvested and disrupted by sonication in buffer A (50 mM Tris-HCl, pH 8.5, 400 mM NaCl and 5% glycerol), and the protein was purified using a Ni chelating column (GE Healthcare) and HiLoad 16/60 Superdex 200 (GE Healthcare). The purified PRS1 protein with a His_6_ tag (LEHHHHHH) at the C- terminus was suspended in buffer A and stored at −80°C for all analysis.

### Site-directed mutagenesis

The plasmids of all mutants were generated from the recombinant PRS1 pET22b(+) vector by site-directed mutagenesis (TaKaRa MutaBEST Kit) and were expressed and purified as described for PRS1.

### Crystallography

Crystals of the PRS1 protein and its mutants suitable for X-ray diffraction were grown in 1.9–2.1 M ammonium sulfate, 1 mM magnesium chloride and 0.1 M sodium citrate (pH 4.0–4.3) using the hanging drop vapor diffusion method at 22°C for 3 days. X-ray diffraction data were collected at 100 K with a cryoprotectant [1.9 M ammonium sulfate, 1 mM magnesium chloride and 0.1 M sodium citrate (pH 4.2), and 25% glycerol] on beamline 17U of the Shanghai Synchrotron Radiation Facility at the Shanghai Institute of Applied Physics, Chinese Academy of Sciences (detector: marccd). Data were processed using HKL2000 and programs in the CCP4 package. Phase determination was performed by molecular replacement using MOLREP in the CCP4 package with the human PRS1 D52H mutant (PDB entry 4F8E) as the initial search model. The final refinement was carried out with refmac5. All crystal structures of PRS1 and its mutants have been deposited into the RCSB Protein Data Bank under accession codes 3S5J, 4LYG, 4LZN, 4LZO, 4M0P and 4M0U. Figures were prepared using PyMOL (http://www.pymol.org/).

### Electron microscopy sample preparation and data collection

Negative stained specimens of PRS1 and PRS1 incubated with different substrates or inhibitors were preserved as previously described[19]. The AMPNPP was purchased from Jena Bioscience and the rest of the reagents (including R5P and ADP) were obtained from Sigma Company. For each sample, approximately 3 μL protein or protein complex was applied to a glow-discharged carbon-coated 400-mesh Cu EM specimen grid. Then, the sample was stained by 0.7% (*w/w*) uranyl formate. We imaged tilted (−55°) and untilted (0°) pairs of PRS1 particles under low-dose condition using a FEI Tecnai F20 microscope (200 kV accelerating voltage, ∼0.6–0.8 μm underfocus) with an FEI Eagle CCD camera at 62,000× magnification. The original pixel size was 1.77 Å per pixel and two-fold pixel-averaged to 3.54 Å per pixel. Particles were selected using the TiltPicker software[20].

### Three-dimensional reconstruction

We obtained the 3D reconstruction using the random conical tilt (RCT) method[20]. In total, we have approximately 4000 single particle tilt-pair images for PRS1. We initially analyzed the 2D images using the Sparx package[21] and then used the resulting averages to run iterative alternating rounds of supervised multi-reference alignment and classification as well as reference-free alignment with Spider[22]. We used Chimera to view all 3D structures[23].

### Sequence analysis

To define the consensus residues involved in substrate binding in all 3 PRS classes [3,4,24], we analyzed the sequences of human PRS1, PRS2 and PRS3, mouse PRS1, *Spinacia oleracea* KPRS1 and KPRS3, *Escherichia coli* KPRS, *Bacillus subtilis* KPRS, *Zea mays* PRS, *Thermoplasma volcanium* KPRS and *Methanocaldococcus jannaschii* KPRS using Weblogo [25,26].

### Enzymatic activity assays

All enzyme activity assays were assayed by determining AMP formation using a method similar to that used in the published paper[27]. The final kinetic constants were extracted by nonlinear fitting to the Michaelis-Menten equation or the Hill equation using the program Origin 7.5.

### MD simulation

MODELLER[28] was used to build the full-length model of D65N, E43T, Q133P, A87T, M115T mutants and WT using their crystal structures respectively. Starting from each structure model, a MD simulation was carried out with a parallel implementation of the GROMACS-4.5.5 package[29], using the CHARMM27 force field[30]. The MD simulation procedure was described as following. The periodic boundary condition (PBC) with a dodecahedron box type was used, with the minimal distance of 1.2 nm between the solute and the box boundary. TIP3P[31] water molecules were added into the box. The steepest descent method was used for the energy-minimization of the system until the maximum force on any atom was less than 1000kJ/(mol.nm). Na^+^ ions were added to the system to neutralize the net negative charge of the system by replacing water molecules with the most favorable electrostatic potential. The final system was energy minimized using the steepest descent followed by the conjugate gradient method until the maximum force on any atom was less than 400kJ/(mol.nm). Verlet integration scheme[32] was used with a 2 fs time-step. A 100 ps equilibration simulation with positional restraint was firstly performed, using a force constant of 1000 kJ mol^−1^ nm^−2^. The initial atomic velocities were generated according to a Maxwell distribution at 310 K. The following production run was 100 ns long. The simulation was performed in a constant NPT ensemble, and the system was coupled to a temperature bath of 310 K through use of an velocity rescaling thermostat[33]. The pressure was adjusted to 1 bar with a relaxation time of 0.5 ps, and the compressibility was 4.5×10^–5^ bar^−1^[34]. Covalent bonds were constrained using the LINCS algorithm[35]. The cutoff distances for the Coulomb and van der Waals interactions were chosen to be 0.9 and 1.4 nm, respectively, and the neighbor list was updated every 10 fs. The long-range electrostatic interactions were treated by the PME algorithm[36], with a tolerance of 10–5 and an interpolation order of 4.

## Results and Discussions

### Conformational changes between the known PRS structures

**Fig 1:**
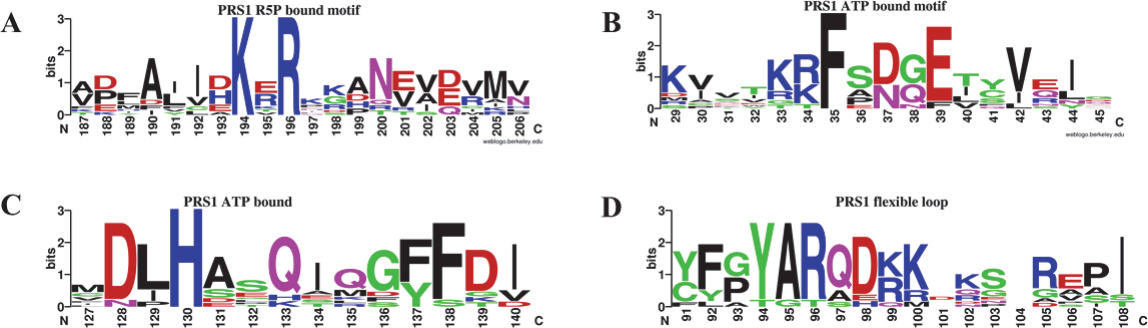
The weblogos of the conserved residues. *(A-D)* The logos highlight the similarities and differences amongst the three PRS classes: class I, II and III. The logos demonstrate the overall conservation of the substrate-bound motifs.

**Fig 2:**
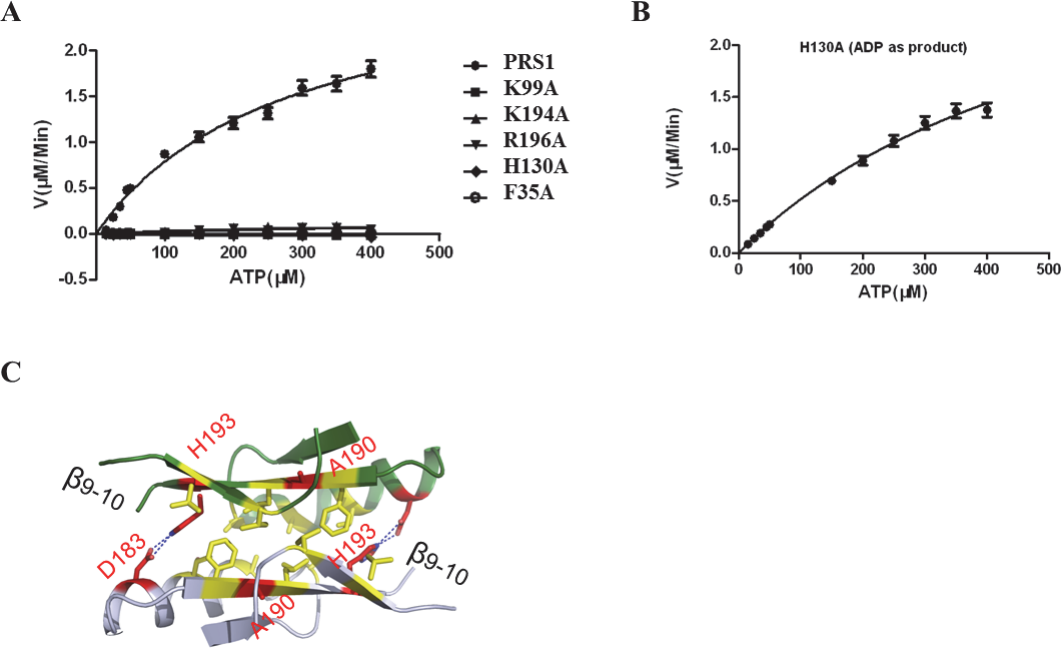
Enzyme assays and 4 mutations located in the β9–10 strands. *(A)* Enzyme assays of PRS1 and its mutants. *(B)* The enzyme assay of the H130A mutant displays no AMP product but produces ADP as its catalytic product. *(C)* The D183H, A190V, H193Q and H193L mutations are located in the antiparallel super flexible β9–10 strands. Error bars were calculated from the mean values, N = 3.

The crystal structure of the human PRS1-SO_4_^2−^ complex was determined using the MR method to 2.0 Å resolution. More information about the 2.0 Å resolution structure could be found in the supplementary materials (S1, S2 Figs. and S1 Table). There are two manifest conformational changes in the PRS structures when all the known PRS structures were compared together (S1 and S2 Video). The first one is the β9–10 strands (S1 Video). In the human PRS1-SO_4_^2−^ complex structure, the β9–10 strands from Arg^196^ to Val^202^, with missing electron density, are possibly structurally and functionally similar to the same region in the *Thermoplasma volcanium* PRS-R5P complex[37]. We also found that the highly conserved residues Lys^194^ and Arg^196^ (located between the β9–10 strands) exist among all 3 classes of PRS (Fig. 1A). We infer that the β9–10 strands might be responsible for the substrate R5P transported to its pocket when superposed with the *Thermoplasma volcanium* PRS and its substrate complexes (PDB code: 3LPN and 3MBI) (S1 Video). To test this hypothesis, the two residues were both individually mutated to Ala (K194A and R196A) prior to conducting activity assays. As a control, the kinetic constants of human PRS1 are listed in Table 1 with a *K*_*m*_ of 278±43.4 μM and *k*_*cat*_ of 89.0±7.33 sec^−1^. By contrast, each of the two mutated versions shows no detectable activity (Fig. 2A), which implies that both Lys^194^ and Arg^196^ are indispensable for R5P binding. The mutation effects of K194A and R196A are also consistent with the data in *Bacillus subtilis* K197A and R199A [38]. Moreover, the reported D183Q, H193Q and H193L mutations[7], which cause a PRS1 gain-of-function phenotype, provide indirect *in vivo* evidence supporting this viewpoint as any of the 3 mutations can destroy the salt bridge between Asp^183^ and His^193^ and induce a higher flexibility of the β9–10 strands (Fig. 2C). Furthermore, the more hydrophobic A190V substitution, with PRS1 super activity, is also located in the β9–10 strands (Fig. 2C). Consequently, the β9–10 strands should be responsible for transferring R5P to its binding pocket.

**Table 1:** Enzyme assays of PRS1 and one mutant.

	*K_m_* (μM)	*k_cat_* (s^−1^)	*k_cat_/K_m_* (μM^−1^ s^−1^)
**WT**	278±43.4	89.0±7.33	0.32
**A130(ADP)**	405±71.2	84.1 ±1.25	0.21

The second motif with conformational change, termed as the flexible loop (from Ile^89^ to Ile^107^), exists among all known PRS structures (S2 Video). Structural comparisons between human PRS1 and the *Thermoplasma volcanium* PRS-ATP complex structure (PDB code: 3LPN) help to confirm the ATP binding pocket, of which the key residues involved are highly conserved (Fig. 1B, C). To confirm the importance of the conserved residues, Phe^35^ (located in the β2–3 strands) and His^130^ were both single mutated to Ala (F35A and H130A) for activity assays. However, although the H130A or F35A mutation does not display normal AMP activity (Fig. 2A), the H130A shows an ADP activity with a higher *K*_*m*_ of 405±71.2 μM and a similar *k*_*cat*_ of 84.1 ±1.25 sec^−1^ (Fig. 2B and Table 1). These results indicate that the Phe^35^ and His^130^ are essential for ATP binding. Additionally, the H130A mutation results indicate the residue’s importance in guarantee of the correct product.

According to structure and sequence analysis, the human Lys^99^ is the residue that is homologous to *Bacillus subtilis* PRS Arg^104^ (Fig. 1D). When analyzing the *Thermoplasma volcanium* PRS-ATP complex (PDB code: 3LPN), we inferred that the flexible loop is able to sweep across the ATP α-β phosphodiester bond using Lys^99^ and cut it into AMP and PPi moiety (S2 Video). To test this hypothesis, we mutated Lys^99^ to Ala^99^ and found no detectable activity (Fig. 2A). Consequently, in combined with the possible orbit crossing the substrate ATP, we propose that the flexible loop is likely essential for ATP cutting.

### Crystal structure of E43T, D65N, A87T, M115T and Q133P mutants

To determine the structural effects of all 16 mutations, we aimed to solve all of those structures. For the 7 gain-of-function mutations, we solved and published the structure of the D52H mutant [27]. We also solved the structures of 5 of the 8 loss-of-function mutations [5,9]. We determined the crystal structures of the E43T, D65N, A87T, M115T and Q133P mutations at a resolution of 3.0 Å, 2.14 Å, 3.3 Å, 2.11 Å and 2.74 Å, respectively, with 2 molecules in each asymmetric unit (S1 Table).

**Fig 3:**
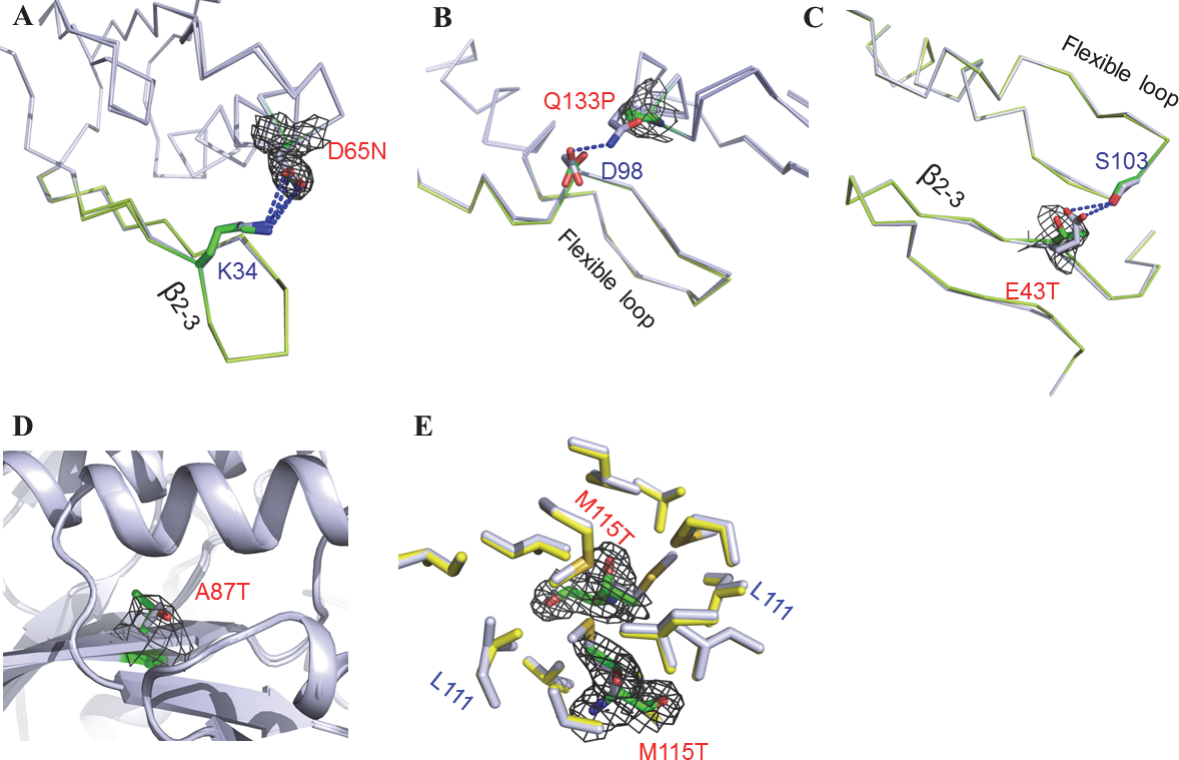
Structural differences between PRS1 and its mutants. *(A-E)* Crystal structure superposition between PRS1 and its mutants. The mutant residue is colored green with the gray Fo-Fc map contoured at 2.5-σ in the image. The antiparallel β2–3 strands and the flexible loop are colored lemon.

**Fig 4:**
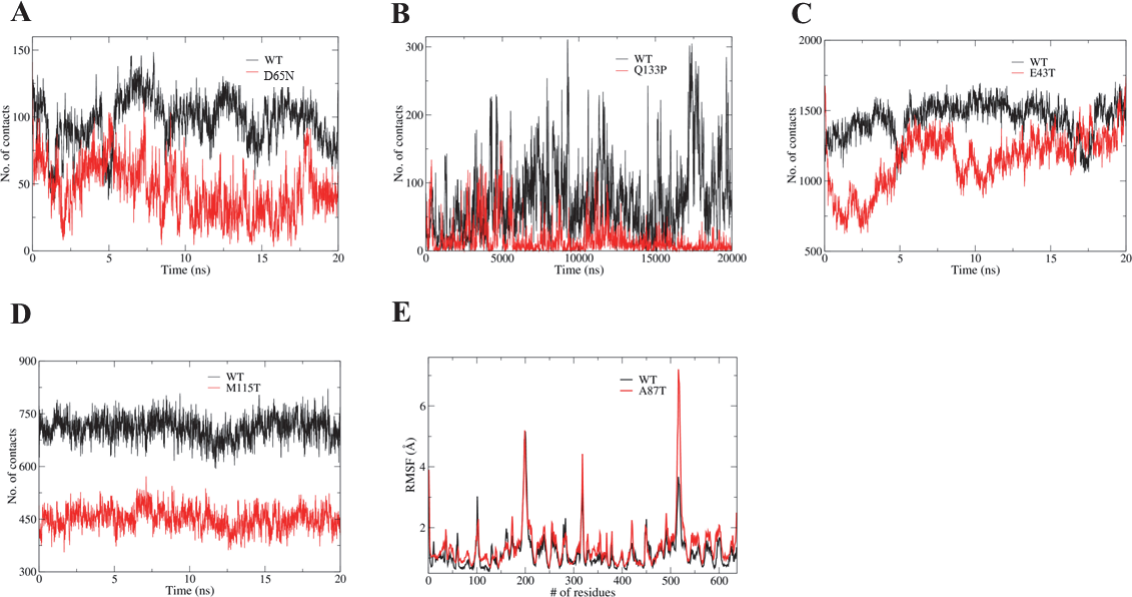
MD simulation results of the five mutants and the WT PRS1. *(A)* Contact numbers of both Asp^65^ in WT (black line) and Gln^65^ in D65N (red line) with the ATP binding motif β2–3. *(B)* Contact numbers of both Gln^133^ in WT (black line) and Pro^133^ in Q133P mutant (red line) with the catalytic flexible loop.*(C)* Contact numbers between the ATP binding motif β2–3 and the catalytic flexible loop for both the WT (black line) and the E43T mutant (red line). *(D)* Contact numbers of both Met^115^ in WT (black line) and Thr^115^ in M115T mutant (red line) with residues that are involved in the interactions with them in the crystal structures, respectively. *(E)* RMSF of all residues in both WT (black line) and A87T mutant (red line).

Overall, the structures of all the five structures are highly similar to that of the wild type PRS1 with RMSD listed in S2 Table. Each mutation causes minor structural change s but destroys or reduces apparent interactions. In the D65N structure, the salt bridge between Asp^65^ and Lys^34^ in the PRS1 structure is replaced by a hydrogen bond between Asn^65^ and Lys^34^, which might reduce the restraint of the ATP binding motif β2–3 (Fig. 3A). In order to confirm this, we performed the MD simulation (Fig. 4A), which indicates that there are fewer contacts between the Gln65 in D65N mutant and the ATP binding motif β2–3. In the Q133P structure, the Gln^133^ replaced by Pro^133^ destroys the hydrogen bond interaction between Gln^133^ and Asp^98^ in the PRS1, which might reduce the restraint of the catalytic flexible loop (Fig. 3B). The MD simulation result indicates that there are fewer contacts between the Pro^133^ in Q133P mutant and the catalytic flexible loop (Fig. 4B). In the low resolution E43T structure, the Glu^43^ replaced by Thr^43^ might destroy the hydrogen bond interaction between Glu^43^ and Ser^103^, which might reduce the restraint of both the ATP binding motif β2–3 and the catalytic flexible loop (Fig. 3C). It is confirmed by the MD simulation (Fig. 4C). In the A87T and M115T structures, the mutations of hydrophobic residues decrease the hydrophobic character of both Ala^87^ and Met^115^ (Fig. 3D-E). In the PRS1 structure, Ala^87^ is located in the hydrophobic core of the N-terminal domain, whereas Met^115^ is located at the hydrophobic A-B dimer interface. Consequently, the M115T mutation might affect the A-B dimerization interface (Fig. 4D), whereas the A87T mutation might slightly affect the secondary structural elements of the N terminal domain (Fig. 4E).

### PRS1 structures visualized by negative stain EM

**Fig 5:**
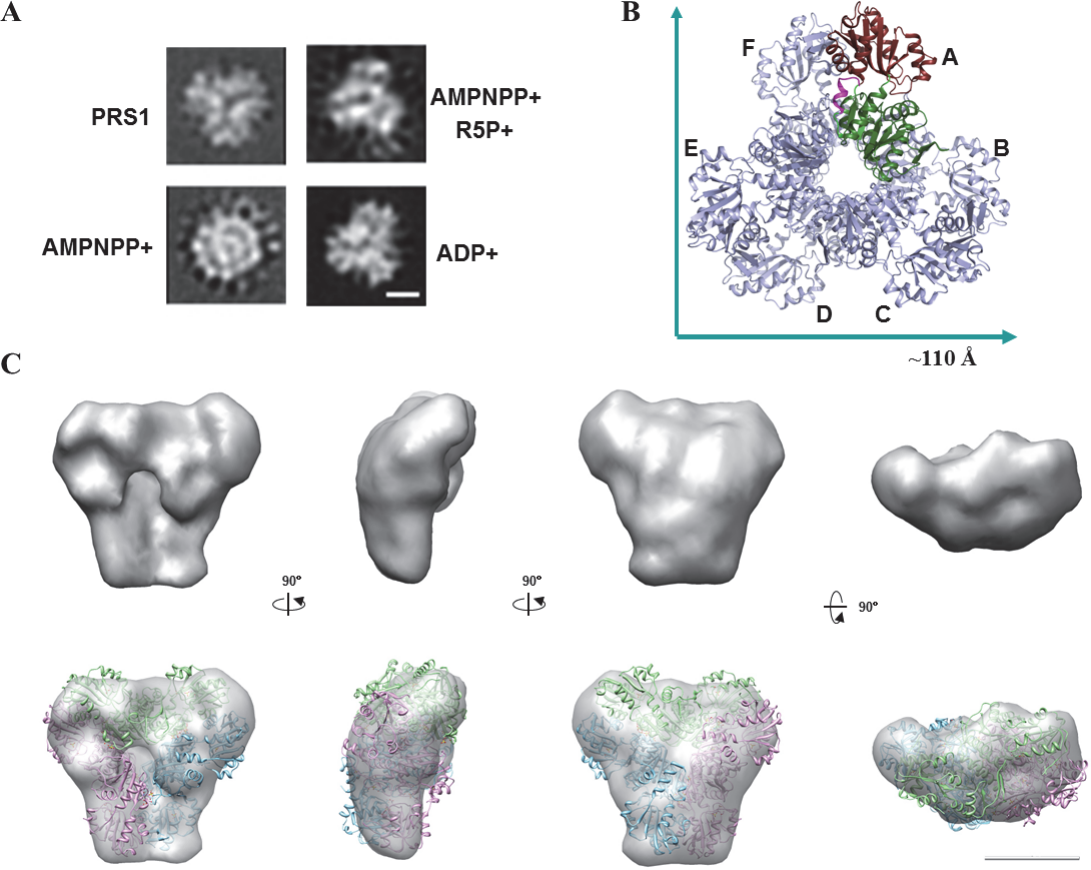
Structures of the PRS1 in different states. *(A)* Negative stain EM of PRS1 and its complexes with AMPNPP, AMPNPP and R5P, and ADP. The four 2D averages (with scale bar, 50 Å) were identified through reference-free alignment and the classification of EM images of single particles preserved in the negative stain. *(B)* Hexamer subunit arrangement according to crystallography symmetry operation. *(C)* The 3D-EM structure of PRS1 with its hexamer crystal structure docked. The 3 different colors represent the three “head to head” dimmers like displayed in Fig. 1B. The figures shows different views of the PRS1 hexamer (PDB:3S5J), which was docked to the 3D EM structures. Scale bar, 50 Å.

Our extensive efforts to cocrystallize PRS1 with ATP, AMPNPP, AMPNPP and R5P, or R5P have not been successful. We then studied the structures of PRS1 in the AMPNPP (ATP analogous), AMPNPP and R5P, ADP and *apo* states by the negative stain EM method (S3 Fig.). The 2D images from the classification of particles show the typical top view of each state (Fig. 5A), demonstrating that the *apo* form of PRS has a 3-leaf clover structure which is similar to the crystal structure of the PRS1 hexamer (Fig. 5B). However, the PRS1-AMPNPP complex shows a distinct conformation with a sunflower shaped edge and a more compact center, which implies that the binding of ATP activates the enzyme from resting state to excited (Fig. 5A, S4A–B Fig.). The PRS1-ADP complex also displays as a similar 3-leaf clover structure (Fig. 5A, S4C–D Fig.). The PRS1-AMPNPP-R5P complex displays more complicate conformations (S4E–F Fig.), which might be caused by different stages of catalysis reaction. We are working on to get more evidences and will give a deep discussion on these complicated conformations in the future.

The negative stain EM of the AMPNPP, AMPNPP and R5P, ADP and the *apo* states confirm the three-leaf clover or sunflower-like structures (Fig. 5A). Our findings are not only consistent with those class I PRSs [4,18] but also provide direct evidence of almost all the primary substrates or inhibitor states of the hexamer. By the way, during sample preparations, the addition of the R5P substrate alone to PRS1 caused it to precipitate immediately; therefore, we could not obtain the 2D structure of the PRS1-R5P complex state. However, incubation with both R5P and AMPNPP did not result in PRS1 precipitation. This finding suggested that PRS1 could only be stabilized with ATP analogs or both R5P and ATP together.

We then reconstructed the 3D- EM structure of the PRS1 *apo* form, which displays the 3-leaf clover structure that matches the PRS1 hexamer model derived from the crystal structure, according to the crystallography symmetry operation (Fig. 5B-C, S3E Fig.). In the present work, we reconstructed 3D structures of PRS1 in its *apo* state, which demonstrates a three-leaf clover conformation (Fig. 1C). According to the crystallography symmetry operation, the PRS1 crystal structure appeared as a three-leaf clover like hexahydric ring that matched the *apo* state of the PRS1 3D-EM structure. Based on the EM results and the crystal structure, the functional hexamer might be the most efficient functional form.

### Molecular basis for disease-associated mutations of PRS1

**Fig 6:**
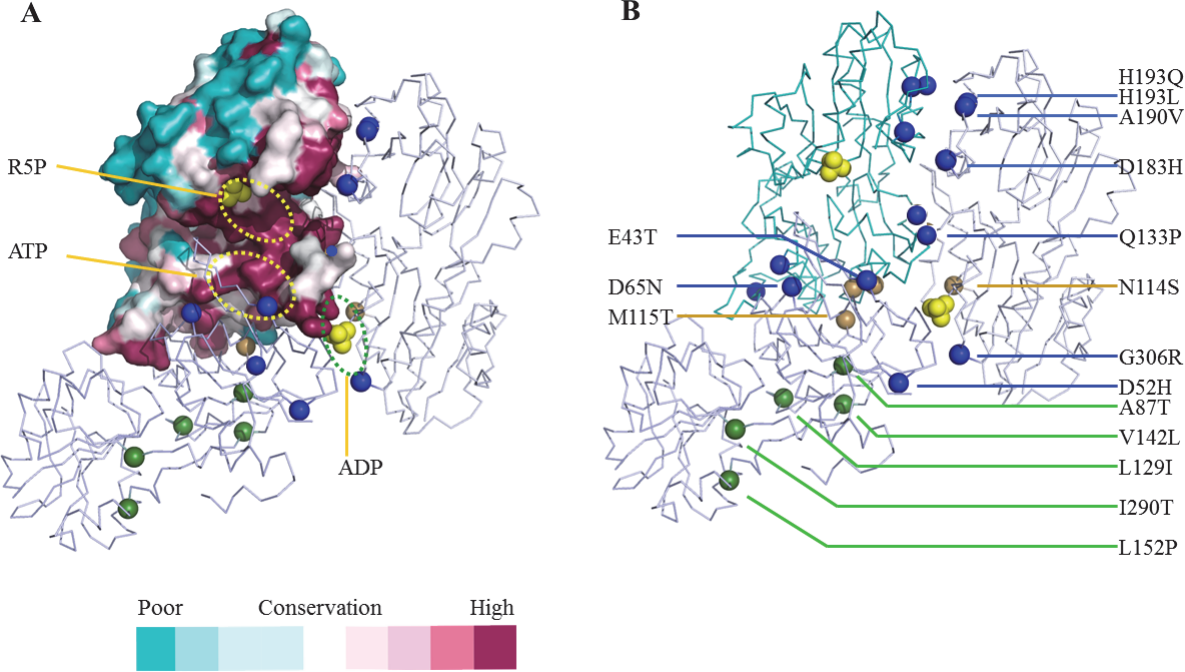
The surface conservation among PRS proteins and the distribution of human PRS1 missense mutations associated with disorders. *(A)* Only subunit A is shown for the surface conservation analysis of the PRS proteins. The dashed ovals represent different substrate pockets as indicated. The balls represent the different disorders associated with the residues. *(B)* The distribution of 16 different PRS1 missense mutations. The brown balls represent the residues distributed at the interfaces. The blue balls represented those near substrates or inhibitor binding pockets. The green balls represent those distributed in the inner parts of N- or C-terminal domains.

The surfaces of active centers and interfaces are highly conserved according to our surface conservation analysis (Fig. 6A). All 16 missense mutations [5,7–10] are distributed along interfaces or the inner part of the domains (Fig. 6B). However, the pathogenesis of these mutations is still unknown. According to the 5 solved and one published D52H [27] structures, none of these mutations changes the overall structure of PRS1.

In the present work, nearly 56% of the mutations disrupt the substrates binding motifs or the catalytic flexible loop. The low activity D65N mutation affects the ATP-binding β2–3 strands directly (Fig. 3A). The D183H, A190V, H193L and H193Q mutations, which are located within the β 9–10 strands, might improve R5P transfer and therefore display as superactivity (Fig. 2F). The D52H mutant reduces ADP binding ability [6,27] and consequently shows as superactivity. The G306R mutant, near the ADP binding pocket, might reduce the inhibitor effect and therefore exhibits a loss-of-function phenotype (Fig. 6A). The Q133P mutant influences the catalytic flexible loop due to the loss of hydrogen bonds, consequently results in a loss of function (Fig. 3B). The E43T mutant affects both the ATP binding motif and the catalytic flexible loop and therefore displays as low activity (Fig. 3C). Nearly 13% of the mutations affect dimer interfaces directly, such as N114S[39] and M115T (Fig. 3E). The N114S mutant decreases the hydrogen bond interaction whereas the M115T mutant reduces the residue’s hydrophobic interactions around the interface (Fig. 6B). These 2 mutations do not affect the protein structure, but may slightly decrease the protein’s aggregation states, thereby affects the protein’s regulation or catalytic efficiency. Approximately 31% of these mutations have hydrophobic side chains and low solvent accessibilities, such as A87T, L129I, V142L, L152P and I290T (Fig. 6B). These mutations might disrupt the secondary structure at given sites, and may interfere with the hydrophobic interaction of the secondary structure elements, which stabilize the protein quaternary structure.

In summary, our findings provided structural insight into the ATP and R5P binding and catalytic key site of the hexamer. Additionally, the analysis of these mutants might provide a framework for future therapy or drug design investigations.

## Supporting Information

S1 Fig Crystal structure of the PRS1. *(A)* Monomer structure of human PRS1. The N-terminus is colored green, the C-terminus is colored ruby and the C terminal 3_10_ helix 2 is colored purple. The linkers between domains are colored yellow. *(B)* The dimer in the one asymmetry structure with subunit A colored as in A and subunit B colored silvery white. The 2*Fo-Fc* density map of 3 SO_4_^2−^s with sigma 1.0 is colored dark blue.
(TIF)

S2 Fig Secondary structure compositions of PRS1. Secondary structure compositions of PRS1.
(TIF)

S3 Fig EM structure of PRS1. *(* ***A***) A typical micrograph of PRS1 preserved under negatively stained (Scale bar corresponding to 50 nm). *(* ***B***) Representative of single particles of PRS1. The scale bar corresponds to 100 Å. *(* ***C***) Two-dimensional (2D) EM analysis of PRS1. The scale bar corresponds to 100 Å. *(* ***D***) Two-dimensional (2D) EM analysis of PRS1 in three variable conformations. The scale bar corresponds to 50 Å. *(* ***E***) Three-dimensional (3D) reconstructions of PRS1 in three variable conformations. The scale bar corresponds to 50 Å.
(TIF)

S4 Fig EM structures of PRS1 with ligands. *(* ***A***) A typical micrograph of PRS1 with AMPNPP preserved under negatively stained. *(* ***B***) Representative of single particles of PRS1 with AMPNPP. *(* ***C***) A typical micrograph of PRS1 with ADP preserved under negatively stained. *(* ***D***) Representative of single particles of PRS1 with ADP. *(* ***E***) A typical micrograph of PRS1 with AMPNPP and R5P preserved under negatively stained. *(F)* Two-dimensional EM analysis of PRS1 with AMPNPP and R5P to show complicate conformations.
(TIF)

S1 Table Data collection and refinement statistics. (DOC)

S2 Table The value of r.m.s.d of structure comparisons between human PRS1 and the related proteins. (DOC)

S1 Video The first manifest conformational change is the β9–10 strands. The movie was generated using PDB 3LPN and 3MBI. The ligand in the movie is R5P. The movie was made using Chimera.
(MPG)

S2 Video The second manifest conformational change is the flexible loop. The movie was generated using PDB 3S5J, 1DKU, and 1DKR. The ligand in the movie is ATP. The movie was made using Chimera.
(MPG)
