# Supplementary figures and images for: Crystal and EM Structures of Human Phosphoribosyl Pyrophosphate Synthase I (PRS1) Provide Novel Insights into the Disease-Associated Mutations
Source: PLoS One. 2015 Mar 17;10(3):e0120304. doi: 10.1371/journal.pone.0120304 (PMC4363470; doi:10.1371/journal.pone.0120304)

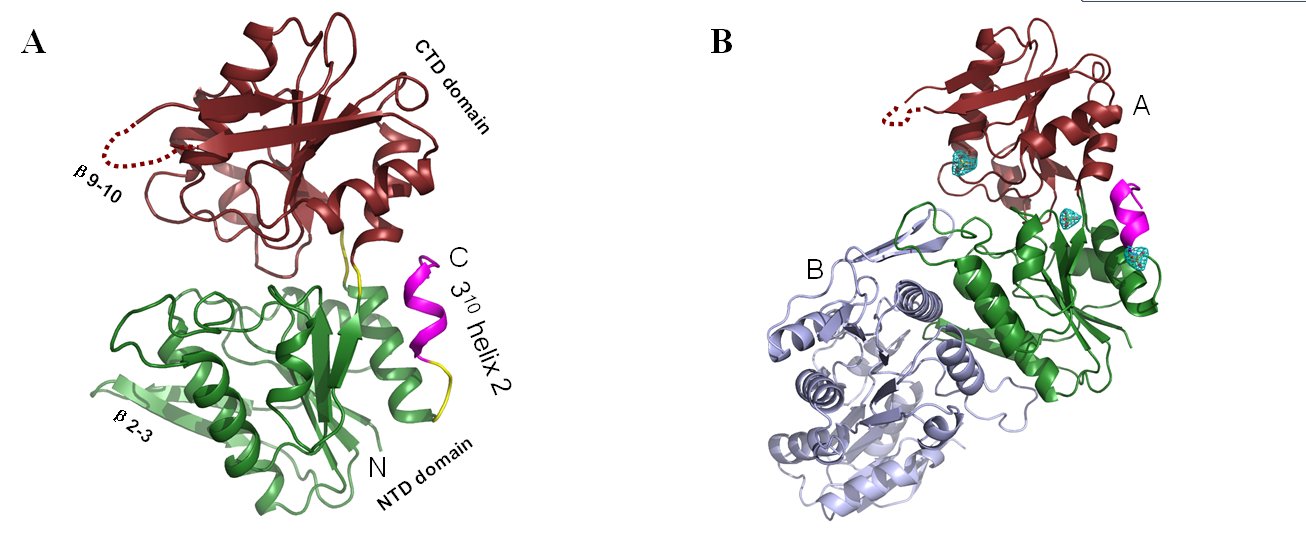

Supplement: S1 Fig — (A) Monomer structure of human PRS1. The N-terminus is colored green, the C-terminus is colored ruby and the C terminal 310 helix 2 is colored purple. The linkers between domains are colored yellow. (B) The dimer in the one asymmetry structure with subunit A colored as in A and subunit B colored silvery white. The 2Fo-Fc density map of 3 SO4 2−s with sigma 1.0 is colored dark blue. (TIF) [file pone.0120304.s001.tif]

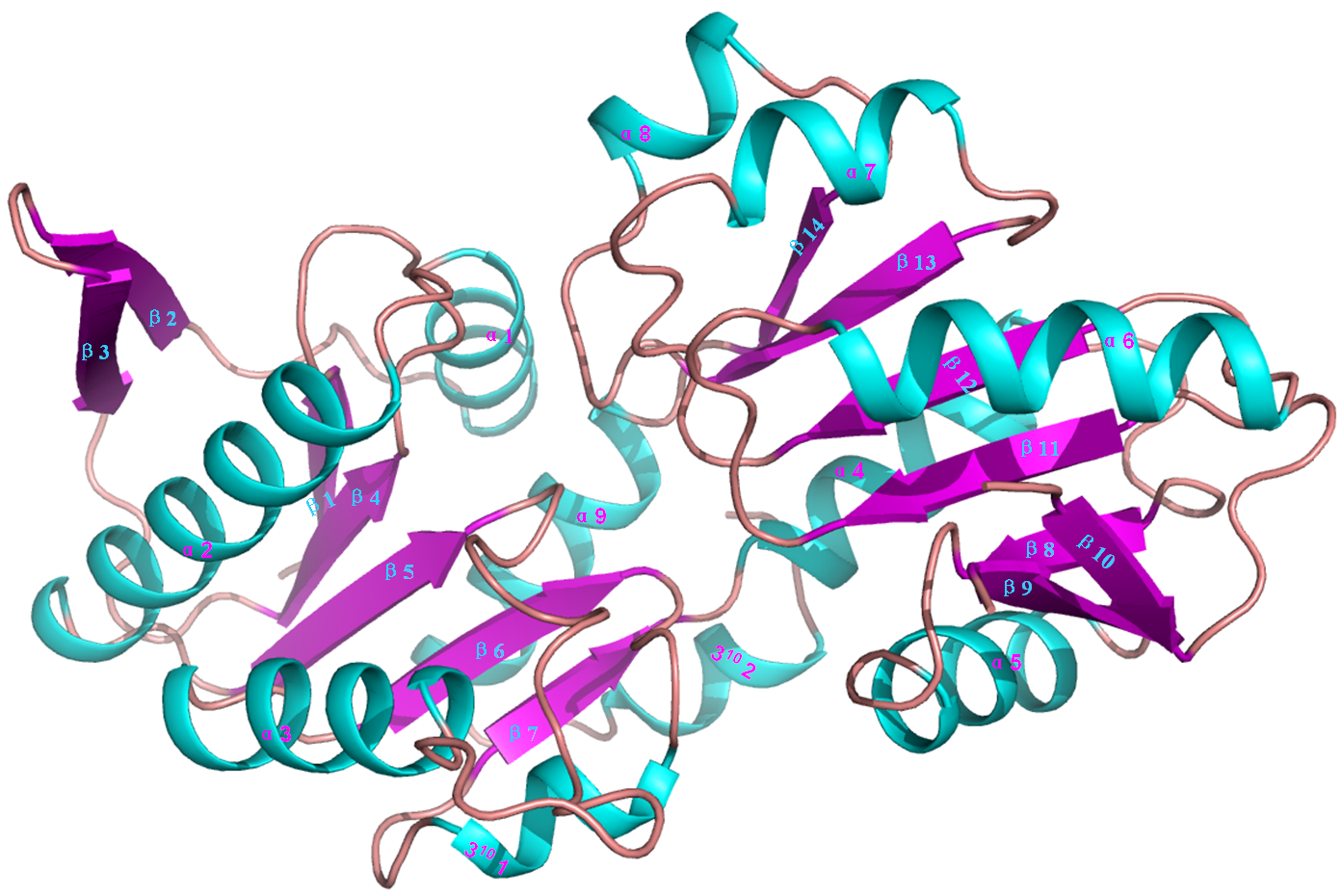

Supplement: S2 Fig — Secondary structure compositions of PRS1. (TIF) [file pone.0120304.s002.tif]

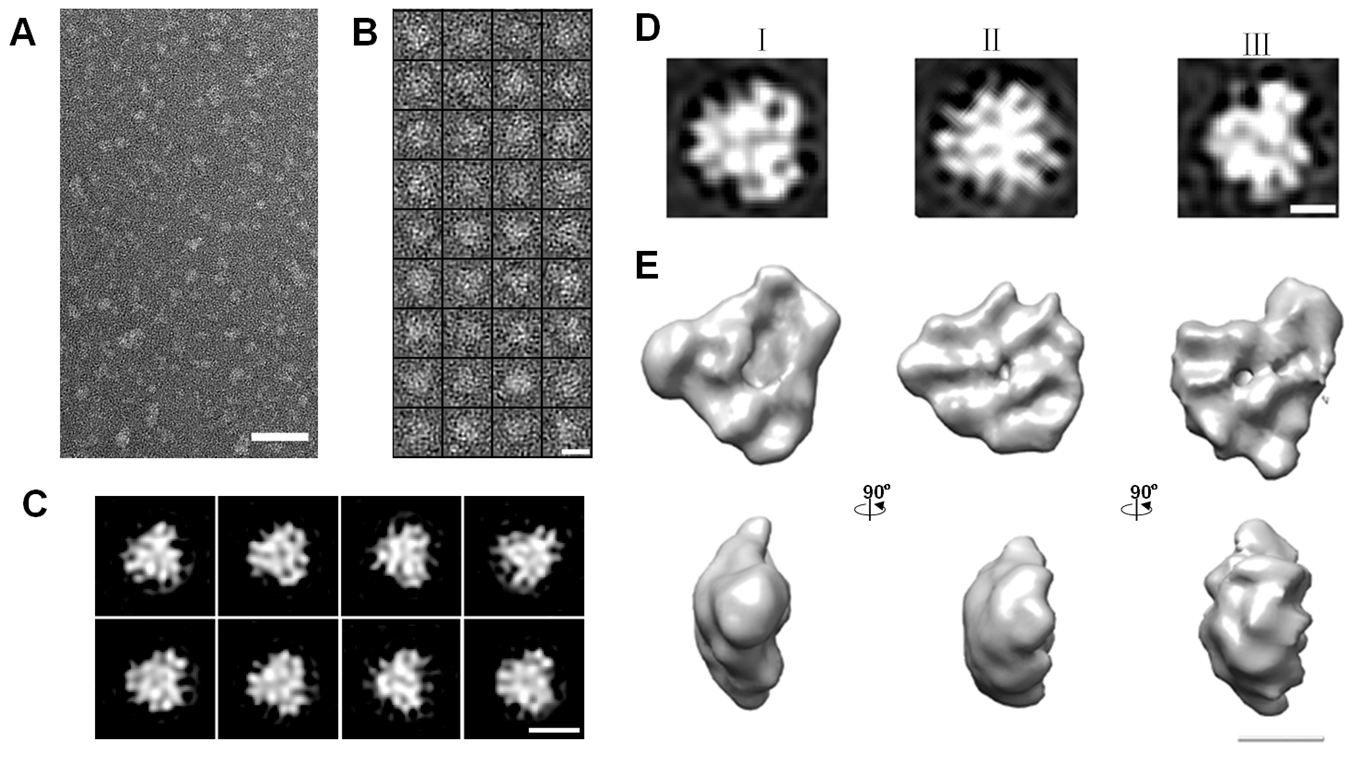

Supplement: S3 Fig — ( A) A typical micrograph of PRS1 preserved under negatively stained (Scale bar corresponding to 50 nm). ( B) Representative of single particles of PRS1. The scale bar corresponds to 100 Å. ( C) Two-dimensional (2D) EM analysis of PRS1. The scale bar corresponds to 100 Å. ( D) Two-dimensional (2D) EM analysis of PRS1 in three variable conformations. The scale bar corresponds to 50 Å. ( E) Three-dimensional (3D) reconstructions of PRS1 in three variable conformations. The scale bar corresponds to 50 Å. (TIF) [file pone.0120304.s003.tif]

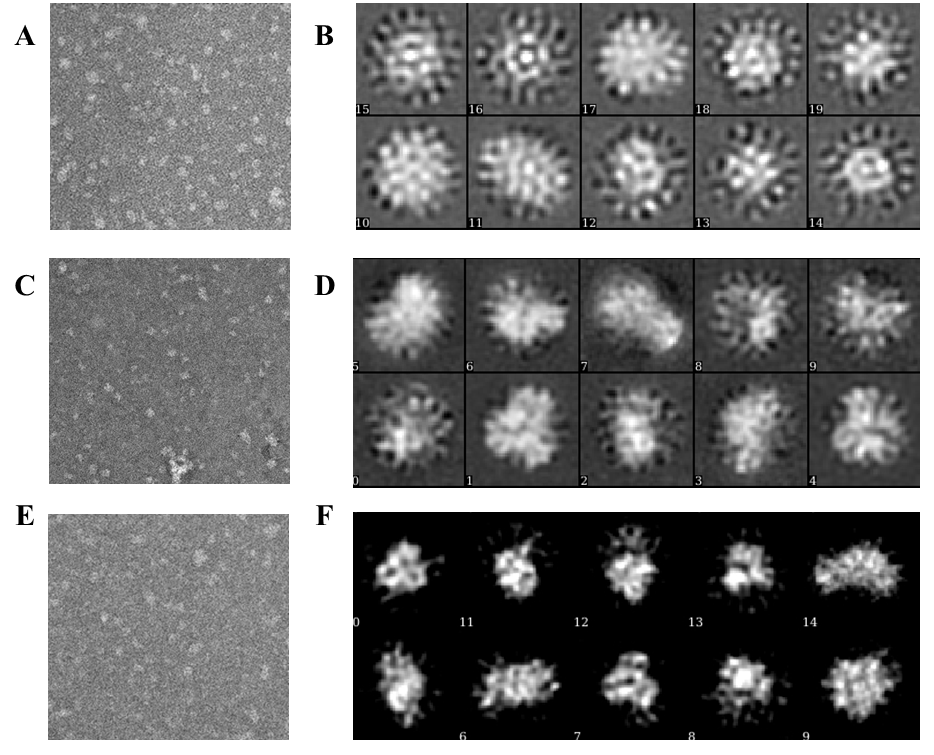

Supplement: S4 Fig — ( A) A typical micrograph of PRS1 with AMPNPP preserved under negatively stained. ( B) Representative of single particles of PRS1 with AMPNPP. ( C) A typical micrograph of PRS1 with ADP preserved under negatively stained. ( D) Representative of single particles of PRS1 with ADP. ( E) A typical micrograph of PRS1 with AMPNPP and R5P preserved under negatively stained. (F) Two-dimensional EM analysis of PRS1 with AMPNPP and R5P to show complicate conformations. (TIF) [file pone.0120304.s004.tif]
