# Supplementary material for: Crystal and EM Structures of Human Phosphoribosyl Pyrophosphate Synthase I (PRS1) Provide Novel Insights into the Disease-Associated Mutations
Source: PLoS One. 2015 Mar 17;10(3):e0120304. doi: 10.1371/journal.pone.0120304 (PMC4363470; doi:10.1371/journal.pone.0120304)
Supplement: S1 Table — (DOC) [file pone.0120304.s005.doc]

**Table S1** **Data collection and refinement statistics.**

|  | PRS1 | E43T | D65N | A87T | M115T | Q133P |
| --- | --- | --- | --- | --- | --- | --- |
| **Data collection** |  |  |  |  |  |  |
| Space group | H3 | | | | | |
| Cell dimensions |  |  |  |  |  |  |
| *a*, *b*, *c* (Å) | 170.54,170.54,61.78 | 169.50,169.50,61.87 | 170.78,170.78,61.70 | 170.40,170.40,62.41 | 170.08,170.08,61.62 | 169.88,169.88,61.76 |
| α, β, γ (°) | 90, 90, 120 | 90, 90, 120 | 90, 90, 120 | 90, 90, 120 | 90, 90, 120 | 90, 90, 120 |
| Resolution (Å) | 40.23 – 2.02  (2.07- 2.02) * | 48.90-3.00  (3.05 - 3.00) | 85.40 − 2.14  (2.18 - 2.14) | 34.20-3.30  (3.36-3.30) | 49.10-2.11  (2.15-2.11) | 34.00-2.74  (2.79-2.74) |
| *R*_sym_ or *R*_merge_ | 9.0(47.0) | 11.1(55.3) | 6.4(42.9) | 15.5(41.6) | 10.0(47.8) | 8.9(38.8) |
| *I* / σ *I* | 18.0(2.5) | 12.9(1.8) | 22.6(2.1) | 14.0(2.4) | 17.5(2.4) | 13.7(1.9) |
| Completeness (%) | 99.8(98.8) | 98.5(97.0) | 95.4(66.1) | 100.0(100.0) | 99.9(98.7) | 92.7(83.1) |
| Redundancy | 4.8(3.0) | 4.1(2.9) | 5.1(3.2) | 5.8(5.1) | 5.8(2.8) | 3.6(2.4) |
|  |  |  |  |  |  |  |
| **Refinement** |  |  |  |  |  |  |
| Resolution (Å) | 40.23 – 2.02 | 48.90-3.00 | 85.40 − 2.14 | 34.20-3.30 | 49.10-2.11 | 34.00-2.74 |
| No. reflections | 41673 | 12462 | 33412 | 9535 | 36190 | 15356 |
| *R*_work_ / *R*_free_ | 21.25/25.43 | 25.55/30.18 | 22.42/26.32 | 26.33/31.18 | 17.14/19.07 | 25.31/29.77 |
| No. atoms |  |  |  |  |  |  |
| Protein | 4680 | 4721 | 4667 | 4684 | 4669 | 4668 |
| Ligand/SO_4_^2-^ | 30 | 25 | 30 | 30 | 30 | 20 |
| Water | 178 | 0 | 69 | 0 | 52 | 15 |
| *B*-factors |  |  |  |  |  |  |
| Protein | 35.6 | 40.5 | 55.1 | 36.5 | 38.8 | 49.8 |
| Ligand/SO_4_^2-^ | 43.1 | 57.9 | 69.2 | 32.1 | 51.5 | 72.6 |
| Water | 39.0 | 0 | 48.1 | 0 | 36.9 | 42.4 |
| R.m.s. deviations |  |  |  |  |  |  |
| Bond lengths (Å) | 0.008 | 0.010 | 0.007 | 0.008 | 0.009 | 0.010 |
| Bond angles (°) | 1.149 | 1.277 | 1.066 | 1.141 | 1.144 | 1.159 |
| PDB entry | 3S5J | 4LYG | 4LZN | 4LZO | 4M0P | 4M0U |

*Values in parentheses are for highest-resolution shell.
