# Supplementary material for: Crystal and EM Structures of Human Phosphoribosyl Pyrophosphate Synthase I (PRS1) Provide Novel Insights into the Disease-Associated Mutations
Source: PLoS One. 2015 Mar 17;10(3):e0120304. doi: 10.1371/journal.pone.0120304 (PMC4363470; doi:10.1371/journal.pone.0120304)
Supplement: S2 Table — (DOC) [file pone.0120304.s006.doc]

**Table S2 The value of r.m.s.d of structure comparisons between human PRS1 and the related proteins.**

| Species and protein name | PDB code | Amino acids range | NO of Cα | Ligand | r.m.s.d(Å)^a^ | Identity(%) |
| --- | --- | --- | --- | --- | --- | --- |
| *t.v* KPRS | 3LPN | M1/I284 | 228 | ATP | 1.780 | 26 |
| *t.v* KPRS | 3MBI | M1/D285 | 227 | R5P | 1.681 | 26 |
| *h* PRS1 | 2HCR | N3/P317 | 273 | AMP | 0.153 | 100 |
| *b.a* OPRT | 3OSC | P63/V93,I106/D173 | 59 | PRPP | 0.978 |  |
| *b.s* PRS | 1DKU | N8/F315 | 250 | ADP | 0.684 | 47 |
| *h* APRT | 1ZN7 | Y60/E180 | 42 | R5P/Mg^2+^ | 1.201 |  |
| s.s UPRT | 3G6W | R34/T46,N72/R105,K115/A192 | 36 | PPi | 1.338 |  |
| *b.s* PRS | 1DKR | 1S/F315 | 265 |  | 0.736 | 47 |
| *m.j* KPRS | 1U9Y | 1M/284L | 246 |  | 2.604 | 27 |
| H PAP39 | 2C4K | 7G/350R | 271 |  | 0.820 | 43 |
| H PAP41 | 2JI4 | 19G/368N | 264 |  | 0.843 | 45 |
| *h* PRS2 |  | | | | | 95 |
| *h* PRS3 |  |  |  |  |  | 94 |
| E43T | 4LYG | N3/P317 | 585 |  | 0.205 | 99.7 |
| *D65N* | 4LZN |  | 594 |  | 0.159 | 99.7 |
| A87T | 4LZO |  | 589 |  | 0.209 | 99.7 |
| M115T | 4M0P |  | 591 |  | 0.224 | 99.7 |
| Q133P | 4M0U |  | 581 |  | 0.367 | 99.7 |

^a^ r.m.s.d is short for root mean square deviation.

t.v: Thermoplasma volcanium

h: Human

b.a: Bacillus anthracis

b.s: Bacillus subtilis

s.s Sulfolobus solfataricus

m.j: **Methanocaldococcus jannaschii**

PRS : phosphoribosyl pyrophosphate synthetase

OPRT: Orotate phosphoribosyltransferase

APRT: Adenine Phosphoribosyltransferase

UPRT: Uracil phosphoribosyltransferase
